# Association between the number of adopted implementation strategies and contextual determinants: a mixed-methods study

**DOI:** 10.1186/s12913-022-08736-2

**Published:** 2022-12-13

**Authors:** Aizhan Karabukayeva, Larry R. Hearld, Reena Kelly, Allyson Hall, Jasvinder Singh

**Affiliations:** 1 Department of Health Services Administration, School of Health Professions, University of Alabama at Birmingham, 3201 1st Avenue North, Birmingham, AL 35222 USA; 2 Department of Health Administration & Policy, School of Health Sciences, University of New Haven, West Haven, CT 06516 USA; 3 Division of Immunology and Rheumatology, School of Medicine, University of Alabama at Birmingham, Birmingham, AL 35222 USA

**Keywords:** Implementation strategy, ERIC, CFIR, Systematic lupus erythematosus, Decision-aid, Implementation

## Abstract

**Background:**

The successful implementation of evidence-based innovations to improve healthcare delivery often requires a well-planned strategy to support their use. With a greater recognition of the importance of an implementation process, researchers have turned their attention to implementation strategies and their customization to target specific organizational barriers and facilitators. Further, there is a paucity of empirical evidence demonstrating the link between implementation determinants and the number of selected implementation strategies. The purpose of this mixed methods analysis is to examine how formatively assessed barriers and facilitators to implementation relate to the number and type of implementation strategies adopted to address context-specific factors.

**Methods:**

A mixed methods evaluation that included 15 rheumatology clinics throughout the United States that were planning for implementation of an evidence-based shared decision-making aid for patients with lupus. Quantitative data consisted of a count of the number of implementation strategies used by a clinic. Qualitative data collection was guided by the Consolidated Framework for Implementation Research (CFIR) and relied upon semi-structured interviews with 90 clinic members between November 2018 and August 2019.

**Results:**

Using the CFIR, we found that local clinic factors (Inner Setting Domain) resulted in different perceptions about Planning and Executing the DA (Process Domain); these domains were most likely to distinguish between the number and type of implementation strategies adopted by the clinics. In contrast, Intervention characteristics, Individual Characteristics, and the Outer Setting did not differentiate between the groups with different numbers of implementation strategies. The number and type of chosen strategies were not those associated with the context-specific factors.

**Conclusions:**

Findings show that, despite recognition of the value of customizing implementation strategies for the contexts in which they are applied, they are too often chosen in a manner that fail to adequately reflect the diverse settings that may present unique factors associated with implementation. Our findings also highlight the importance of the inner context – both in terms of structural characteristics and existing work processes – as a driving factor for why some organizations select different numbers and types of implementation strategies.

**Supplementary Information:**

The online version contains supplementary material available at 10.1186/s12913-022-08736-2.

## Background

The successful implementation of evidence-based innovations (EBIs) to improve healthcare delivery requires a well-planned strategy to change ‘the way we do things around here’ [1, 2]. This is because evidence from innovation research does not indicate easy translation into practice, especially if fit with the local context has not been addressed [3, 4]. Literature has shown that the outcomes of implementation vary by organization: contextual factors such as leadership support, capacity, culture, and resources can greatly affect how readily new EBIs are adopted [5–7]. However, contextual factors such as organizational culture and climate are difficult to change and are often beyond the direct control of implementers. It may be more useful to think about how cultural elements can be leveraged and “it would probably be most effective to focus culture change efforts as narrowly as possible” [8]. Accordingly, researchers have turned their attention to implementation strategies, defined as specific tools or techniques (e.g., educational programs, incentives) used to improve uptake of EBIs [9–11]. They are social interventions targeting “various properties of interventions that make them more or less amenable to implementation” [10]. Studies suggest that implementation strategies are most effective at promoting the use of EBIs when they are customized to the unique barriers and facilitators in a setting [12–17].

Despite recognition of the value of customizing implementation strategies for the contexts in which they are applied, strategies are too often designed and applied in a standardized manner and fail to adequately reflect the diverse settings with different implementation determinants [18–20]. For example, Bosch et al. [21] examined 22 quality improvement studies and found that the selected implementation strategies did not always match identified implementation barriers. Even if the prospective barrier analysis was conducted, only a few authors explicitly mentioned the methods used to translate the results into tailored strategies. Thus, the literature indicates that in many cases no explicit theoretical or evidential rationale is provided for a chosen strategy [22, 23]. Instead, it appears that in the absence of knowing what works, implementers use many different approaches [24]. Consequently, we know relatively little about how organizations choose different implementation strategies in real world settings and the factors that may drive these choices [25–27].

Another aspect of implementation strategies often considered is the number of strategies (i.e., single or multifaceted). Historically, the assumption seems to have been that the use of multiple implementation strategies, i.e., a multifaceted or multicomponent approach to implementation, is more effective in promoting adoption of EBIs. For example, a study of U.S. Department of Veterans Affairs clinics working to implement evidence-based HIV treatment found that implementers used an average of 25 (plus or minus 14) different implementation strategies [28]. Likewise, a review by Bacci et al. found that most studies in community pharmacist patient care services utilized more than one implementation strategy, with a mean of 6.5 strategies (range, 1–36 strategies) [29]. Despite these examples and the assumption underlying them, there remains a paucity of empirical evidence on the motivations and factors underlying the decisions that result in more or less implementation strategies. The purpose of this mixed methods study is to identify factors that might prompt organizations to choose different numbers and types of implementation strategies. The analysis is based on a formative evaluation of 15 rheumatology clinics throughout the United States that have been planning for implementation of an evidence-based shared decision-making aid (DA) for patients with lupus. Insights from the study are important for better understanding how contextual factors shape the number and type of implementation strategies.

### Consolidated Framework for Implementation Research (CFIR)

The identification of contextual determinants was guided by the Consolidated Framework for Implementation Research, while the implementation strategies were informed by a typology of implementation strategies. The CFIR is a widely used framework that is designed to assess potential determinants of implementation within local settings [20]. The CFIR organizes 39 standardized constructs into five major domains. Definitions of these domains and their operationalization in this study are described in Table 1. In this analysis, the 39 constructs and their five parent domains were used to (1) develop a semi-structured interview guide, (2) organize our thematic analysis of contextual determinants that were associated with decisions surrounding choice of implementation strategies.

**Table 1:** CFIR operationalization

CFIR Domains	Description	Operationalization
Intervention Characteristics	Features of the intervention being implemented into a particular setting that might influence implementation	Individualized, evidence-based, patient-centered, computerized lupus decision-aid (DA)
Outer Setting	Economic, political, and social context, typically outside an organization, in which an intervention is implemented	External policies, reimbursement systems, incentives applicable to the rheumatology clinics within the diverse settings (urban vs suburban, number of patients seen, type of clinic, practice type, etc.).
Inner Setting	Structural and cultural context through which the implementation process proceeds	Six of the study clinics are rheumatology-only clinics, while the other nine are multispecialty. Twelve of the study clinics are owned by a university, while only three are not owned by a university. Two of the study clinics have less than 10 members, while nearly half have 31 or more clinic members. On average, the study clinics have 2.27 (SD = 0.25) physical locations where they care for patients. The average number of years of experience in the clinic is 2.28 (SD = 0.25).
Characteristics of Individuals	Impact of individuals on implementation success	Self-efficacy, knowledge, and beliefs about the DA among physicians, pharmacists, administrative director/clinic managers, nurses, medical assistants, patient technicians, front office staff and study coordinators.
Process	Methods by which implementation is accomplished	How clinics would go about using the DA: key people who should get on board with this implementation; current communication mode with patients, how clinics should communicate with lupus patients about the availability of the DA; accessing the DA; how clinic should inform patients about availability of the DA and how to use it.

### Expert Recommendations for Implementing Change (ERIC)

This study utilizes a taxonomy of implementation strategies developed by Powell et al. [30] and the ERIC. It classifies 73 individual implementation strategies into six categories: planning (e.g., assessing readiness and identifying barriers, selecting strategies and getting buy-in), educating (e.g., developing effective educational materials, conducting ongoing training), financing (e.g., altering incentive/allowance structures, facilitating financial support), restructuring (e.g., revising professional roles), managing quality (e.g., developing and organizing quality monitoring systems, conducting audit and feedback, using reminders), and attending to the policy context (e.g., creating or changing credentialing and/or licensure requirements) [30, 31]. Implementation strategies can be utilized individually (i.e., discrete) or combined together to build a tailored multicomponent strategy for implementation [11, 31]. For example, an organization could utilize educational strategies alone (a discrete strategy) or combine training and technical assistance (a multicomponent strategy). Given the range of strategies available and allowances for organizations to choose different discrete strategies in real world settings, different combinations of implementation strategies likely exist among organizations.

Another consideration given varied organizational and implementation contexts is whether implementation strategies should be deployed in a standardized manner or allow organizational stakeholders to customize the strategies or choose which strategies to use in their unique situation. This is a common dilemma in implementation research that seeks to identify effective strategies using rigorous research designs (e.g., randomized control trials) that require some degree of standardization yet recognize the need to consider the important role of context [32]. Standardized strategies are assumed to be effective independent of the context into which an innovation is being introduced. In contrast, customized approaches to using implementation strategies allow organizations to adapt the strategy or choose strategies for their local needs and resources. Customized approaches may be especially valuable in situations where the implementation settings vary substantially and/or where the implementation strategies themselves are delivered by the local organization [11, 17].

## Methods

### Setting

The analysis reported here was part of a larger evaluation of different strategies for implementing the DA [33]. Using a purposive sample, 15 rheumatology clinics were identified through the professional network of the principal investigator and invited to participate based on their geographic distribution throughout the United States and their capacity to meet study criteria (e.g., commitment to use the DA for study duration, ability to recruit minimum number of patients to view the DA).

In this study, we used a combination of standardized and customized implementation strategies to implement the DA designed to educate lupus patients about their treatment options and help them engage in more shared decision making with their physicians. All clinics used standardized implementation strategies that were provided uniformly by the research team (e.g., training on use of DA, designation of a clinic champion and refresher training course). In addition, each clinic could choose from a ‘menu’ of implementation strategies that could be customized to their clinic. These customized implementation strategies were directed to both clinic personnel and patients. Clinic-targeted strategies focused on integrating the DA into existing work processes, while patient-targeted strategies focused on raising awareness and educating patients about the DA. This approach was utilized to provide clinics the flexibility to choose activities that met their unique needs and capabilities. Table 2 provides a list of both the standardized and customized strategies.

**Table 2:** List of implementation strategies

**Standardized Capacity Building Strategies**	**Description and Purpose of Strategy**
Education, coaching, technical assistance	Education - A series of 60-min seminars aimed at all 15 clinics that educate clinic personnel about the DA, including its purpose and content. Coaching - A series of webinars, offered 1–2 months after each sites’ formative evaluation, that described clinic-specific findings of the formative evaluation, and jointly identified the preferred strategies for the clinic. Technical assistance – Ongoing, ad hoc technical support on the use and maintenance of an electronic tablet (e.g., trouble launching the DA, problems navigating screens).
Clinic champion	Designated member of the clinic who is dedicated to supporting the implementation of the DA in the clinic.
Refresher training course	A webinar conducted every 6 months or as needed that describes the implementation strategies and common barriers being confronted across the participating clinics and shares best practices of how these strategies are being deployed
**Tailored, Clinic-targeted Strategies**	**To further integrate the DA into existing work processes**
Patient check-in process revision	Coordinating/training front desk staff to include a reminder of the DA and ensure it does not slow clinic flow.
Feedback on DA use	Discussing current challenges with the DA use and its implementation to keep clinic staff engaged.
Team huddles/clinic meetings	Incorporate the DA as a standing agenda item in team huddles /clinic meetings to increase staff buy-in.
Quarterly newsletters	Disseminating information about on-going challenges and best practices across clinics
**Tailored, Patient-targeted Strategies**	**To raise awareness and educate patients about the DA**
Pre-visit communication	Communication with patients prior to clinic visit via telephone or patient portal introducing the study and providing an electronic link to the DA. The purpose is to make sure the DA does not lengthen the patient visit.
Paper-based version of the DA	Providing patients with PowerPoint slide deck to make sure patients are not overwhelmed with information.
Pamphlets/posters	These information materials are made available in the clinic to increase understanding/interest in the DA.
Video about the DA in the waiting room (e.g., kiosk)	Making additional information materials available to address possible challenges related to language/literacy/understanding/interest.

### Data sources

The analysis reported in this paper relied primarily on two data sources. Qualitative data were collected via semi-structured telephone interviews during the first 6 months of the study, between November 2018 and August 2019. The purpose of these formative interviews was to understand local circumstances surrounding the anticipated implementation of the DA, especially barriers and facilitators to implementation. The interview protocol was constructed using the CFIR technical assistance website (https://cfirguide.org/) and included questions related to all five domains of the CFIR (Additional file 1_Interview guide). These questions were then adapted to fit our study. Further, additional questions were added to the protocol using the subject matter expertise of the study investigators. Three evaluation team members (LH, AH, RK), who were trained in qualitative interviewing, piloted the initial interview protocol with four key informants to assess the clarity of the questions, identify gaps in the interview guide, and improve the flow of the interview. Subsequent interviews were conducted by these same three evaluation team members.

A study coordinator and/or the principal investigator of each clinic selected key informants from a variety of positions within a clinic, including physicians, pharmacists, administrative directors/clinic managers, nurses, medical assistants, patient technicians, front office staff and study coordinators, to assure that we captured a wide variety of perspectives on potential challenges to implementing the DA. The eligibility criteria included being an employee of the clinic and familiar with the clinic’s research and patient care activities. One day prior to each interview, an email was sent to each key informant. The email included a short video that described the DA, a PowerPoint print-out of the DA, and a summary of topics to be discussed during the interview. Each interview began with a verbal informed consent of the potential benefits and risks of participating in the study and their rights as participants. Field notes, written during and after interviews, were included in the project files. The IRB approved the use of verbal informed consent given that the interviews were conducted via telephone. During the consent process, clinic staff were informed that participation was voluntary, and they could withdraw from the study at any time. Overall, 90 interviews were conducted across all 15 clinics, with an average of six key informants per clinic (range 2 to 9). The average duration of the interviews was 34 minutes (range 15 to 60). Table 3 provides details about informants and clinic groupings by number of strategies. All interviews were recorded and subsequently transcribed verbatim, yielding 658 pages of transcribed text. Data saturation was reached when the evaluation team determined that new interviews were not yielding new information.

**Table 3:** Professional roles of key informants and number of implementation strategies adopted by clinics

Clinic	Number of personnel interviewed	Number of Key Informant Types Interviewed				Number of implementation strategies utilized by the clinics (Low = 1–3; Moderate = 4–6; High = 7–8)
		Physicians	Nurses	Other clinical personnel	Administration/Front Office	
Clinic 1	5	1	0	3	1	High
Clinic 2	8	1	0	2	5	Moderate
Clinic 3	5	1	1	0	3	Moderate
Clinic 4	9	3	2	2	2	Low
Clinic 5	4	1	1	1	1	Low
Clinic 6	6	2	0	1	3	Moderate
Clinic 7	9	1	2	1	5	Low
Clinic 8	6	2	1	0	3	Low
Clinic 9	5	2	1	0	2	Low
Clinic 10	2	1	0	0	1	High
Clinic 11	4	2	0	0	2	High
Clinic 12	8	4	1	1	2	Low
Clinic 13	7	1	2	3	1	Low
Clinic 14	6	1	2	0	3	High
Clinic 15	4	2	0	0	2	Moderate

Quantitative data consisted of a count of the number of implementation strategies used by a clinic to implement the DA (range 3 to 11). These data were collected from the 15 clinics as a part of the DA implementation process. Prior to initiating DA use at each clinic (6 months following the start of the study), two members of the research team (JS and LH) met virtually with the implementation team at each clinic. The clinic implementation teams included the site principal investigator (a rheumatologist), clinic champion, nurses, and office staff. The purpose of these virtual meetings was to summarize the perceived barriers to implementing the DA identified during the formative interviews described above [34]. Another purpose was to select implementation strategies that the implementation team believed were feasible in their clinic and would be effective at facilitating implementation of the DA. The study team used the ERIC to identify a candidate set of implementation strategies for each clinic based on its specific barriers. As noted earlier (Table 2), all clinics were given the freedom to choose standardized or customized strategies. The higher number of strategies meant that clinics added more customized strategies. We recorded the implementation team’s initial selection of strategies. Approximately 3 months after initiating the DA at each clinic, we asked the clinic champion to verify these strategies, including adding ones that were subsequently adopted and removing ones that were never used. We used the second verified list in our quantitative analysis.

### Analytic strategy

Qualitative data analysis, consisting of both coding, synthesis, and memo writing, utilized a team of four investigators (LH, AK, RK, AH) to mitigate issues of bias, and thus, provide greater confidence in the coding and analysis process. Transcribed interviews were coded using NVivo 12 Plus (QSR International). Our coding proceeded through two stages and utilized a deductive and inductive approach. As part of the larger evaluation, the four investigators independently coded three different transcripts from the same key informants based on the CFIR constructs/subconstructs which were subsequently mapped to the domains of the CFIR (e.g., relative advantage mapped to intervention characteristics). Kappa statistics were used to assess inter-rater agreement (IRA), with estimates for all five major domains exceeding 0.830. In addition, investigators subsequently met to discuss the source of any remaining coding differences and the lead evaluator (LH) regularly conducted peer debriefing with other members of the research team to minimize potential bias and assumptive coding. Once the differences were reconciled, the 15 clinics were divided among the four team members (range 2–5 clinics per team member) for applying the codes (deductive stage). Further, team members identified emergent themes within the CFIR domains (inductive phase). These themes were then summarized in the form of a written memo for each domain that described key themes and how they were expected to affect implementation of the DA. During kick-off meetings with stakeholders, these memos were shared and discussed to verify accuracy [34].

In the next analytic step, clinics were grouped into three groups: low, moderate, and high number of implementation strategies. The underlying reason for grouping clinics into three groups was to explore whether those that chose just 1–2 strategies (just the standard ones) differed from those clinics that chose 6–8 strategies (added clinic- or patient-targeted strategies). Clinics were assigned to these groups using classical multidimensional scaling (MDS). MDS is an exploratory data reduction technique (similar to factor analysis but reduces cases rather than variables) used to obtain quantitative estimates of similarity among groups of items. Scree plots were used to assess how many dimensions were appropriate, which in our case indicated three dimensions. Given that the input data reflected the number of implementation strategies utilized by the clinics, we labeled these groups: 1. High number of implementation strategies group (HNIS) (10–11 total strategies); 2. Moderate number of implementations strategies group (MNIS) (7–9 total strategies); and 3. Low number of implementation strategies group (LNIS) (4–6 total strategies).

In the final analytic step, we synthesized the themes within each of these strategy groups, wrote three memos (one for each group), and finally compared and contrasted the themes across the groups. Our objective in the final step was to identify distinguishing contextual factors between the three groups with different numbers of implementation strategies.

## Results

Since the purpose of this analysis was to understand what factors might prompt organizations to choose different numbers and types of implementation strategies, the results reported below focus on the key differences between the three groups. Most of the distinguishing factors we identified related to the Inner Setting and Process Domains. While not discussed in detail below, common contextual determinants are listed in Table 4.

**Table 4:** Overview of CFIR constructs linked to common themes across groups

*CFIR constructs*	*Themes*	*Quotes*
**Intervention Characteristics**		
*Relative advantage: perception of the advantage of implementing the intervention* versus *an alternative solution.*	*Empowering tool*	*“I feel like there was a really good balance between all the scientific information, but it is user-friendly... it’s not dumbed down too much either” (Clinical nutritionist/research associate, HNIS)* *“I think it’s a great tool to get patients to understand more about their disease and help making the decision along with the clinician by having them understand and all the details and side effects of medications and choices that are available. So, all of that is helpful for the patients to understand and if you see it on a formal slide or information sheet, they will believe in it more and make it easier for us to move forward.” (Physician, MNIS)* *“…it is very useful for a person who may need more help in talking with their doctor about their care. It breaks down their risk in a way that might help them understand what they really need to know about that specific treatment.” (Clinical research coordinator, LNIS)*
*Design Quality and Packaging: perceived excellence in how the intervention is bundled and presented.*	*Time and duration as a barrier*	*“I’m a little bit worried about its length and then the attention span for people for doing this within the clinic. It seems like the length may be more appropriate for review at home, kind of an on-demand type of thing.” (Physician, HNIS)* *“They [patients] disregard the late policy…may arrive 1.5–2 hours late to their appointment and still be seen here. Now you are taking an extra 20 minutes as well [to view the DA]. I don’t know whether our providers are going to be on….” (Registered nurse, MNIS)* *“So, I only have about 20 minutes per patient for a follow-up, and some of that time is often spent checking patients in, and I’m booked every 20 minutes. If the decision aid takes 20 minutes for the patient to read, then the only way I could see it working is to send the patient home with that information and then do a follow-up. Or alternatively, have them come back in. The problem is a lot of our patients come from a significant distance, so getting them to come back in within a short turnaround time, both for their logistics and our schedule, is often not convenient.” (Physician, LNIS)*
*Complexity: perceived difficulty of implementation*	*The added workload of technology*	*“The study is not a problem because I’ll be able to coordinate how many patients are coming in a day. So, if we have five iPads, we won’t schedule more than five appointments within the same hour or two. But, if it’s going to be implemented as a regular thing in the clinic, our supply would be an issue and another for front desk staff to worry about distributing, collecting. Because it’s going to have to be charged, and if someone drops it or it breaks, we must send it out for repairs, things like that.” (Study coordinator, HNIS)* *“…we must find a place to lock them [iPad] up when we leave and there should be a station to charge them. So, those kinds of things we have to look into.” (Dynamic scheduler service rep, MNIS)* *“There would be a need a specific person who is in charge of making sure they [tablets] were returned. I don’t know if it would be the front desk or the nurses, but someone would need to be in charge of making sure it doesn’t get lost or something.” (Research coordinator, LNIS)*
**Outer Setting**		
*Patient Needs and Resources: extent to which patient needs are accurately known and prioritized by the organization*	*Implementation challenges of a diverse patient mix*	*“The person, whoever is implementing the study, should have specific training about how to introduce this in a way that a patient is more likely to say yes than no. Our patient population is fairly educated, highly educated and professional, and in many cases they’re not here for half a day. They’re here in and out. So that is going to be the challenge in our clinic.” (Physician, HNIS)* *“I think it would be accepted well, depending on patient though. We have a pretty research active lupus population. We have a registry study that is involved with the patient, so I just feel like the association of research is familiar with a lot of patients that come to our clinic, so they would be more accepting of looking at the decision aid” (Study coordinator, MNIS)* *“We see a lot of indigent patients, and those with lower socioeconomic backgrounds. I feel like this is going to be hard for not all of them, but some of them [to] really understand. I think it’s a little overwhelming. We have some really sick patients and to have this on top of them not feeling good, or they have their kids with them and can’t concentrate, just stuff like that I could see interfering with it.” (Research nurse, LNIS)*
**Inner Setting**		
*Structural characteristics: age, maturity, or size of the organization* *Compatibility: integration into existing work processes*	*Fitting in a workflow*	*“We are going to have to figure out how to integrate in our already complicated workflow…and ultimately, I think that is going to be the biggest barrier. How do we implement this without disruption to their primary tasks?” (Physician, HNIS)* *“I think one problem is going to be its effect on the workflow, because we’re an extremely busy clinic, and if the patient shows up late which they often do, how is it going to impact our office flow? (Physician, MNIS)* *“…the concerns I have are just workflow and diagnosis…if the doctors get bought in and they want their patients to have it, they’re going to have to find a way to pre-identify patients because we wouldn’t have the bandwidth to go snooping through patients’ charts. It would have to be something that was ideally in place before the patient got here and all we would have to do is give it to them and maybe explain what it is.” (Administrative supervisor, LNIS)*
*Culture: norms, values, and basic assumptions of a given organization*	*Openness to change, conditional upon conducive structure and culture*	*“I think people [clinic members] would be pretty open to it. People would not be afraid to try something new. Some of the older staff who have been there longer may be a little bit more ingrained, but I would say it is mostly younger staff that is here. They would be open to changes.” (Physician, MNIS)* *“The clinic is very open to trying new things. For instance, one of our studies that we’re working on is the influence of incorporating virtual reality to reduce stress in patients with autoimmune disease. That is just an example of us using a novel technology as an experimental intervention to see if it can help our patients. So, we’re very open to it.” (Clinical research coordinator, HNIS)* *“We are open to change. We’ve been changing rules. I’ve been here six years, during that period they changed computer systems and we adapted to it. I don’t think it’s the employees against the change. I mean, providers might not be against it, but I don’t have that type of conversation with them. But that’s where I feel like we’ll have an issue. (Certified clinical medical assistant, LNIS)*
**Characteristics of Individuals**		
*Knowledge and Beliefs about Intervention: individual staff knowledge and attitude towards the intervention*	*Positive attitude*	*“It’s definitely a novel approach. It could be very helpful. There could be a few barriers to it. It’ll take a little bit of doing and trying, but it’s definitely something that could be implemented.” (Physician assistant, HNIS)* *“I think that once the tool is introduced and is set up, I don’t anticipate at this point that they need to do any changes in the clinic.” (Triage RN, MNIS)* *“Well, the opportunities lie in the enthusiasm we all have in making the care of lupus patients more streamlined, efficient and of quality…. So, I think that it’s fine. I don’t see any issues.” (Director of the clinic/physician, LNIS)*
*Self-efficacy: an individual’s belief in his/her capabilities to execute the implementation*	*Clinical personnel experience with research and improvement*	*“I think we are in a unique situation; we are pretty flexible in our clinic where we can start to implement these things relatively straightforward. It is not getting without growing pains, but nonetheless, I don’t see a huge hurdle in terms of implementing that once we are all familiar with the factual assessment and everything.” (Physician assistant, HNIS)* *“For quality improvement, one initiative that we have implemented is we have what we call a friends and family forum. That was very well received, and I initiated that because we’re in triage and when we’re on the phone and call people, we have to have that form in place so we can give information that’s appropriate. When there is a need, or we see an initiative or a project that can be started, it is very well received by our staff. Ideas bounce off each other. That is a positive for our clinic.” (Triage registered nurse, MNIS)* *“I think we have been reasonably successful before. We do sort of have patient clinic questionnaires that we have been implementing for a while and we had to change our infrastructure around that as well….so, that will sort of help us if we have more challenges, but we have been reasonably successful in incorporating changes.” (Physician, LNIS)*
**Process**		
*Engaging: engaging individuals in implementation process*	*Need for buy-in from physicians*	*“We have a lot of different goals in mind at this clinic. It be confusing with what study you want to take priority. So, usually we will focus most of our resources on getting what he [physician] wants to get done first, and then after we finish that, we will be able to handle the auxiliary studies. So, if he sets a priority, we definitely would put most of our time and effort into it to make sure it is done well.” (Clinical research coordinator, HNIS)* *“It’d be better if everyone is on board. Obviously, getting the physicians on board is critical, but I think it’s the nurses, PAs or whoever is framing the patients, and even for that matter, the secretaries at the front. I think they would be the ones first mentioning the decision aid to the patient. So, getting them involved, as well, like the secretaries and the nurses would be pretty important, too, since they’re the people who have the first interactions with the patient when they arrive to the clinic.” (Physician, MNIS)* *“…we really need strong physician engagement. Leadership can only do so much, in my opinion. We really need the physicians and have them lead the implementation. And I think with [physician], he’s been very engaged and he’s very involved in all his patient care. So, we really need someone like [physician] be the spearhead of programs like this. In the past we haven’t done it because of physician engagement, but I think now they’re very engaged and they really want their patients to be more educated for their condition. (Clinic administrator, LNIS)*

### Inner setting domain

#### Structural Characteristics & Compatibility

It is notable that most clinics in the MNIS and LNIS groups were members of larger organizations that had a more formal, centralized structure with sharply drawn functional and professional delineations that resulted in relatively little interaction between the physicians and nurses. Such separation was seen as a challenge to coordinate efforts to identify suitable patients for viewing the DA. Moreover, some members did not strongly identify with the clinic due to technically being employed and managed by the larger organization.

*“The nurses that we have are not specific to our clinic. They will see pulmonary patients, renal patients and be rooming those patients at the same time. It is just a function of how their work is set up that we just do not have a ton of interaction with them.” (Physician, MNIS).*

*“…the physicians have very little say in how the clinic runs. The nurses do not report to us. We do not hire the nurses. We do not hire the support staff. We have no say in the hiring or firing of those individuals.” (Physician, LNIS).*

*“It takes a while to get up to change… kind of give you a sense of the complexity: the front desk people who check in our patients there, they report directly to one supervisor. Once [patients]checked in, they go back to the hallway to be processed and triaged by our nursing MA; those people report to a different supervisor. So, there’s a lot of complexity.” (Physician, LNIS).*

Notably, these differences in Structural Characteristics and Compatibility presented problems for the integration of the DA with existing organizational processes, which ultimately affected how the clinics approached implementing the DA. This issue is described in more detail in the Process Domain (see below).

#### Networks and communications

Groups also differed in how they communicate and involve clinic personnel in decisions. Specifically, HNIS clinics were much more inclusive and proactive in communicating with the entire staff about the purpose and required actions for projects.

*“Whenever anything new is being implemented, we always have a meeting. We have a meeting every week. So, everything that is going on in the office or something new that is coming up is said in that meeting. If something that is happening right then, our supervisor will come right away and let us know what we need to do. We know the events for the month” (Practice office associate, HNIS).*

*“I think [physician] is very good with meeting with the research team. He meets with us at least two to three times weekly, we talk about what we’ve been doing during the week, he presents us with policy changes, studies, ideas. So, as far as communication, it’s usually done in person, and it’s usually done by [physician] himself.” (Clinical research coordinator, HNIS).*

*“We hold a monthly clinic meeting, if it’s something that they need to be aware beforehand, then I send out an email and usually float around clinic and make sure everybody is aware of any changes.” (Clinic manager, MNIS).*

In contrast, interviewees from clinics in the LNIS group believed that communication was patchier and more inconsistent across different members of the clinic, especially front-line administrative staff.

*“We’re not really involved in any of that stuff. I know my doctor does research, but I have no idea what he does or where he goes.” (Medical assistant, LNIS).*

#### Culture

The groups of clinics also differed with respect to their culture. Clinics from HNIS group reported having more of a personal and dynamic organizational culture, characterized by teamwork and employee collaboration as a critical factor of work environment. On the other hand, the LNIS clinics had more formal, hierarchical cultures with different departments/specialists working independently. Clinics within the MNIS did not have a consistent, dominant culture type.

*“…Everyone is very willing to help, even if you perform work that is not what the provider intended for it to be. Everything is presented in a way that is constructive and not demoralizing” (Clinical research coordinator, HNIS).*

*“We do have rules and things that are in place for safety, but we are a big family and it’s an open-door policy that anybody can come in and ask for anything or give suggestions. So, I would say it’s a little bit of both” (Triage clinic lead, MNIS).*

*“There is a very regimented instruction as to how the clinic is operationalized for a lot of different reasons, not just because of specialty services, but also how many physicians are seeing patients at any given day, what is the volume of the specialty areas…” (Research director, LNIS).*

#### Available resources

Groups also differed with respect to their available resources. Interviewees from HNIS clinics did not consistently report resource deficiencies as a barrier, while interviewees from the MNIS and LNIS felt they lacked resources to implement the DA. Notably, however, the types of resources differed between these two groups. Interviewees from MNIS clinics felt they lacked the human resources to reliably implement the DA, mostly due to turnover.

*“There is a motivation to do it. But there are time constraints, and we are also very short staffed right now in terms of physicians. So, that is limiting how much we are able to do.” (Physician, MNIS).*

*“That would be my biggest thing that would impede it. The other thing would be that because we do not consistently always have the same two people in the clinic, because we have people who float through, that might probably be a little bit of a barrier too.” (Registered nurse, MNIS).*

In contrast, interviewees from LNIS clinics also described a lack of human resources, but in this case (and as noted above) because those resources were not in the clinic’s control. In addition, clinics from LNIS group felt they lacked the physical space that would ideally be available to implement the DA.

*“I am removed from the clinic in a sort of sense that I’m not actually a clinic employee. I’m an employee of the university. As I’m placed under the University…. I don’t directly answer to the clinic.” (Research director, LNIS).*

*“The only problem I am concerned about is at any given time there can be three to four rheumatologists and there is a shared space for five clinics all together. So, we don’t have dedicated rooms just for Rheumatology, it is shared. I am concerned if it is up 20 minutes and the staff needs to get in another patient, am I going to be able say “hey can I borrow a room?” and I don’t want a patient to feel rushed, but space is kind of a premium in the clinic.” (Director of the clinic/physician, LNIS).*

### Process domain

#### Planning

All groups discussed the importance of clinical staff to be engaged and educated from the start for greater buy-in and planning for DA implementation. However, MNIS and LNIS clinics placed a greater emphasis on the need for planning, especially for opportunities to adapt the implementation process, due to overlapping priorities and focus on generating income.

*“Just to make sure that everybody is aware and trained on it. So, anybody working with [physician], they should understand that we need a time for the patient to fill this out. We all should be on the same page because that’s always a big thing, we might be told something at the front desk that maybe the nurses aren’t aware of. So, just making sure everybody understands the process, each other’s roles, and understands how it’s going to help the patient. That would be the biggest thing for me.” (Dynamic scheduler service rep, MNIS).*

*“I would like it to be very clear in exactly what needs to be done and how it’s going to be done. I don’t want to be doing it blindly, because we would probably be the one to initiate it with the patient since we are the face they see first. So, if we know what we’re doing, then we’re good.” (Medical assistant, LNIS).*

#### Executing (intersection with inner setting: Structural Characteristics & Compatibility)

Clinics in the LNIS group were more likely to report several inner setting challenges to implementing the DA. In particular, clinics in this group were concerned about the logistics of implementing the DA, including their ability to pre-identify eligible patients and schedule them to view the DA. These issues were especially concerning when the clinics had a centralized scheduling system and corresponding lack of dedicated front office staff, which would result in additional work for other staff and/or more serious work process redesign.

*“So, regarding returning patients, the front desk isn’t even going to know, ‘oh, this is returning lupus patient’, just going through the check-in process. So, I guess that would fall more to the nurse and the doctor as far as like bringing it up on the front end.” (Administrative assistant, LNIS).*

*“It’s a very complex administrative structure. It takes a while to change …and to give you a sense of the complexity, the front desk people who check in our patients, they report directly to one supervisor. So, there’s a lot of complexity there to get things done.” (Chief of rheumatology, LNIS).*

## Discussion

The purpose of this study was to empirically assess whether different types of clinic contextual factors may drive different numbers of implementation strategies while trying to implement an evidence-based DA for patients with lupus. Using the CFIR, we found that factors pertaining to the Inner Setting and Process Domains were most likely to distinguish between the groups with different number of implementation strategies adopted. In contrast, Intervention characteristics (i.e., the DA), Individual Characteristics, and the Outer Setting did not differentiate between the groups. In some ways, this pattern of findings is not surprising. Clinics arguably differed the most with respect to their inner settings, with a diverse range of sizes, culture, and decision-making authority, all of which can play a significant role in successful implementation [35, 36]. All clinics in the LNIS group had structural challenges such as limited resources (i.e., physical space and staff), which had the effect of creating busier clinics with fewer opportunities for choosing a greater number of implementation strategies. In addition, clinics in the LNIS group had more hierarchical, formalized structures, and in contrast to the HNIS group, the LNIS and MNIS were multispecialty clinics and members of academic medical centers. Other research has found that clinic environments with centralized authority relationships like those found in academic medical centers can impose barriers to engaging in learning behaviors and change efforts [37].

It is also notable that the number of perceived barriers in a clinic was negatively associated with the number of implementation strategies. For example, all clinics from LNIS group selected only one clinic-targeted strategy (quarterly newsletters) and only one clinic from this group selected a patient-targeted strategy (clinic poster about DA). In contrast, nearly all clinics from HNIS selected all available clinic- and patient-targeted strategies. One explanation for this pattern is that the choice of implementation strategies is limited by resource availability. Indeed, our analysis revealed differences between the groups with respect to resources, including and especially human resources. Given that the tailored implementation strategies were primarily delivered by the clinic staff, it is conceivable that such deficiencies may have limited their ability or willingness to take on more strategies. To the extent more (or more customized) strategies are effective at promoting implementation of the DA, such differences may result in patchy, differential implementation patterns across clinics that may undermine the DA’s effectiveness at promoting greater shared decision-making among lupus patients. Future research, however, is needed to ascertain whether more (and more customized) implementation strategies are, in fact, more effective at promoting implementation. It is conceivable, for example, that too many strategies may create confusion among clinic members or patients and undermine their collective effectiveness. Likewise, it is possible that some strategies may compete with each other for resources and attention, suggesting that certain combinations of strategies may be most effective at supporting DA implementation.

While not the primary focus of the analysis, our findings with respect to common themes of contextual determinants are supported in the existing literature [38]. In general, all clinics had positive views of the DA, indicating that it would be a useful tool for patients and a good source of information. Another common facilitator was a general consensus among respondents that clinics were supportive of any initiative or program that was perceived to be beneficial for patients. Almost all clinics indicated time as the main challenge to using the DA, and in doing so, highlighted the DA’s potential for disrupting clinic workflow and adding to the workload of the clinic staff. Consequently, nearly all respondents noted the need to get buy-in at all levels and make sure everyone understood the purpose and intended use of the DA. Likewise, because the clinics were characterized as busy settings with multiple competing demands and priorities, it was important to proactively plan for DA use, for example, by pre-identifying eligible patients (e.g., lupus patients scheduled for the day or week) and providing options of delivering the DA before the appointments (e.g., via website at home or iPad in clinic waiting room). These findings highlight some potential implications for practitioners, which are explored below after discussing the study limitations.

Theoretically, one would expect clinics to select strategies with careful planning to ensure that the strategies would effectively address implementation barriers and facilitators. At the same time, there is a value in performing incremental implementation with ongoing evaluation. The literature shows that implementation strategies generally change over time, adapting to the context [28, 39]. Given the formative stage of our evaluation and the cross-sectional nature of our analysis, it is imperative to receive feedback from the staff and make modifications to the implementation plan.

Furthermore, as noted earlier, the factors that differentiated the groups were largely in the Inner Setting and Process Domains. These are the primary domains included in the CFIR that encompass the work-environment factors and implementation processes. This finding suggests that strategies targeting more malleable aspects of an organization and addressing barriers by improving the fit between the DA and the context might have a greater impact. For example, clinics may be able to change the implementation climate or how patients flow through the clinic and get access to the DA. In contrast, barriers related to things in the Outer Setting may be more intractable. Likewise, changes to the DA in this setting, given how it is constructed and delivered, provide few options for change. And even if they could change the DA, it could potentially undermine the effectiveness of the DA. Thus, clinic leaders might consider creating an implementation climate where employees regard innovation use as a top priority, not as a distraction from or obstacle to the performance of their “real” work.

The findings reported above and the implications discussed next should be interpreted in light of several limitations. First, the study included only 15 clinics, and most of these clinics were located within academic medical centers, each representing unique organizational and community contexts and limiting generalizations to other clinics. Future work could address this limitation by including more clinics, including addressing questions about how to disseminate the DA to new settings. Second, it is possible that responses to our interview questions were biased, with key informants wanting their clinic to appear in a positive light. This can especially be the case with organizational research like ours where key informants may feel added pressure to respond positively due the employment relationship [40]. We attempted to mitigate this issue by capturing a broad range of perspectives from clinical personnel across different roles and contexts. Likewise, there is a possibility of selection bias issues (e.g., clinics are more motivated to change and implement the DA due to a contractual relationship for study duration). To mitigate this issue, we recruited clinics with varied characteristics from a wide-range of geographic areas. Nevertheless, future research could still build on this work and address this shortcoming by conducting site visits to observe the use of the DA in situ. In addition, it is conceivable that our analytic approach introduced biases given the subjective nature of coding and developing themes. While we cannot eliminate these biases, our evaluation adopted several steps to mitigate them 1) including multiple respondents from each site; 2) involving multiple investigators in coding and memo writing; and 3) including member checks, whereby a summary of findings was provided to each clinic for feedback and correction.

## Conclusion

Findings show that, despite recognition of the value of customizing implementation strategies for the contexts in which they are applied, they are too often chosen in a manner that fail to adequately reflect the diverse settings that may present unique factors associated with implementation. Our findings also highlight the importance of the inner context – both in terms of structural characteristics and existing work processes – as a driving factor for why some organizations select different numbers and types of implementation strategies.

## Supplementary Information

**Additional file 1.**

## Data Availability

The datasets used and/or analyzed during the current study are not publicly available due to participant privacy. However, the research team is committed to providing some data if requested by other researchers by email to the PI, Jasvinder Singh (jassingh@uab.edu).

## Abbreviations

EBI: Evidence-based innovations
DA: Decision aid
CFIR: Consolidated Framework for Implementation Research
IRA: Inter-rater agreement
MDS: Multidimensional scaling
HNIS: High number of implementation strategies group
MNIS: Moderate number of implementation strategies group
LNIS: Low number of implementation strategies group

## References

[1] Balogun J, Hailey VH (2008). Exploring strategic change: Pearson Education.

[2] Khalil H. Implementing change in healthcare: evidence utilization. JBI Evidence Implementation. 2015;13(2);41–2. 10.1097/XEB.0000000000000045 26057646

[3] Huijg JM, Crone MR, Verheijden MW, van der Zouwe N, Middelkoop BJ, Gebhardt WA (2013). Factors influencing the adoption, implementation, and continuation of physical activity interventions in primary health care: a Delphi study. BMC Fam Pract.

[4] King DK, Neander LL, Edwards AE, Barnett JD, Zold AL, Hanson BL (2019). Fit and feasibility: adapting a standardized curriculum to prepare future health professionals to address alcohol misuse. Pedagogy Health Promot.

[5] Shaw RJ, Kaufman MA, Bosworth HB, Weiner BJ, Zullig LL, Lee S-YD (2013). Organizational factors associated with readiness to implement and translate a primary care based telemedicine behavioral program to improve blood pressure control: the HTN-IMPROVE study. Implement Sci.

[6] Ober AJ, Watkins KE, Hunter SB, Ewing B, Lamp K, Lind M (2017). Assessing and improving organizational readiness to implement substance use disorder treatment in primary care: findings from the SUMMIT study. BMC Fam Pract.

[7] Haffar M, Al-Karaghouli W, Djebarni R, Gbadamosi G (2019). Organisational culture and TQM implementation: investigating the mediating influences of multidimensional employee readiness for change. Total Qual Manag Bus Excell.

[8] Schneider B, Ehrhart MG, Macey WH. Organizational climate and culture. Annu Rev Psychol. 2013;64(1):361–88. 10.1146/annurev-psych-113011-143809 22856467

[9] Flanagan ME, Ramanujam R, Doebbeling BN (2009). The effect of provider-and workflow-focused strategies for guideline implementation on provider acceptance. Implement Sci.

[10] Proctor EK, Powell BJ, McMillen JC (2013). Implementation strategies: recommendations for specifying and reporting. Implement Sci.

[11] Powell BJ, Fernandez ME, Williams NJ, Aarons GA, Beidas RS, Lewis CC (2019). Enhancing the impact of implementation strategies in healthcare: a research agenda. Front Public Health.

[12] Kwok EYL, Moodie ST, Cunningham BJ, Cardy JEO (2020). Selecting and tailoring implementation interventions: a concept mapping approach. BMC Health Serv Res.

[13] Lewis CC, Scott K, Marriott BR (2018). A methodology for generating a tailored implementation blueprint: an exemplar from a youth residential setting. Implement Sci.

[14] Bunger AC, Powell BJ, Robertson HA, MacDowell H, Birken SA, Shea C (2017). Tracking implementation strategies: a description of a practical approach and early findings. Health Res Policy Syst.

[15] Kilbourne AM, Abraham KM, Goodrich DE, Bowersox NW, Almirall D, Lai Z (2013). Cluster randomized adaptive implementation trial comparing a standard versus enhanced implementation intervention to improve uptake of an effective re-engagement program for patients with serious mental illness. Implement Sci.

[16] Wensing M, Bosch M, Grol R (2010). Developing and selecting interventions for translating knowledge to action. CMAJ..

[17] Oxman AD, Thomson MA, Davis DA, Haynes RB (1995). No magic bullets: a systematic review of 102 trials of interventions to improve professional practice. CMAJ. Can Med Assoc J.

[18] Aarons GA, Hurlburt M, Horwitz SM (2011). Advancing a conceptual model of evidence-based practice implementation in public service sectors. Adm Policy Ment Health Ment Health Serv Res.

[19] Flottorp SA, Oxman AD, Krause J, Musila NR, Wensing M, Godycki-Cwirko M (2013). A checklist for identifying determinants of practice: a systematic review and synthesis of frameworks and taxonomies of factors that prevent or enable improvements in healthcare professional practice. Implement Sci.

[20] Damschroder LJ, Aron DC, Keith RE, Kirsh SR, Alexander JA, Lowery JC (2009). Fostering implementation of health services research findings into practice: a consolidated framework for advancing implementation science. Implement Sci.

[21] Bosch M, Van Der Weijden T, Wensing M, Grol R (2007). Tailoring quality improvement interventions to identified barriers: a multiple case analysis. J Eval Clin Pract.

[22] Davis R, Campbell R, Hildon Z, Hobbs L, Michie S. Theories of behaviour and behaviour change across the social and behavioural sciences: a scoping review. Health Psychol Rev. 2015;9(3):323–44. 10.1080/17437199.2014.941722 PMC4566873 25104107

[23] Hooley C, Amano T, Markovitz L, Yaeger L, Proctor E (2020). Assessing implementation strategy reporting in the mental health literature: a narrative review. Adm Policy Ment Health Ment Health Serv Res.

[24] Proctor E, Hooley C, Morse A, McCrary S, Kim H, Kohl PL (2019). Intermediary/purveyor organizations for evidence-based interventions in the US child mental health: characteristics and implementation strategies. Implement Sci.

[25] Powell BJ, Waltz TJ, Chinman MJ, Damschroder LJ, Smith JL, Matthieu MM (2015). A refined compilation of implementation strategies: results from the expert recommendations for implementing change (ERIC) project. Implement Sci.

[26] Waltz TJ, Powell BJ, Matthieu MM, Damschroder LJ, Chinman MJ, Smith JL (2015). Use of concept mapping to characterize relationships among implementation strategies and assess their feasibility and importance: results from the expert recommendations for implementing change (ERIC) study. Implement Sci.

[27] Baker R, Camosso-Stefinovic J, Gillies C, Shaw EJ, Cheater F, Flottorp S, Robertson N. Tailored interventions to overcome identified barriers to change: effects on professional practice and health care outcomes. Cochrane Database Syst Rev. 2010;(3). 10.1002/14651858.CD005470.pub2 PMC4164371 20238340

[28] Rogal SS, Yakovchenko V, Waltz TJ, Powell BJ, Kirchner JE, Proctor EK (2017). The association between implementation strategy use and the uptake of hepatitis C treatment in a national sample. Implement Sci.

[29] Bacci JL, Bigham KA, Dillon-Sumner L, Ferreri S, Frail CK, Hamada CY (2019). Community pharmacist patient care services: a systematic review of approaches used for implementation and evaluation. J Am College Clin Pharm.

[30] Powell BJ, Waltz TJ, Chinman MJ, Damschroder LJ, Smith JL, Matthieu MM (2015). A refined compilation of implementation strategies: results from the expert recommendations for implementing change (ERIC) project. Implement Sci.

[31] Powell BJ, McMillen JC, Proctor EK, Carpenter CR, Griffey RT, Bunger AC (2012). A compilation of strategies for implementing clinical innovations in health and mental health. Med Care Res Rev.

[32] Mazzucca S, Tabak RG, Pilar M, Ramsey AT, Baumann AA, Kryzer E (2018). Variation in research designs used to test the effectiveness of dissemination and implementation strategies: a review. Front Public Health.

[33] Singh JA, Hearld LR, Hall AG, Beasley TM (2021). Implementing the DEcision-aid for lupus (IDEAL): study protocol of a multi-site implementation trial with observational, case study design. Implement Sci Commun.

[34] Creswell JW, Miller DL (2000). Determining validity in qualitative inquiry. Theory Pract.

[35] Greenhalgh T, Robert G, Macfarlane F, Bate P, Kyriakidou O (2004). Diffusion of innovations in service organizations: systematic review and recommendations. Milbank Q.

[36] Lash SJ, Timko C, Curran GM, McKay JR, Burden JL (2011). Implementation of evidence-based substance use disorder continuing care interventions. Psychol Addict Behav.

[37] Hearld L, Hall A, Kelly RJ, Karabukayeva A, Singh J. Organizational context and the learning and change readiness climate for implementing an evidence-based shared decision-making aid in US rheumatology clinics. J Health Organ Manag. 2021. 10.1108/JHOM-10-2020-0397 34232597

[38] Elwyn G, Scholl I, Tietbohl C, Mann M, Edwards AG, Clay C (2013). “Many miles to go…”: a systematic review of the implementation of patient decision support interventions into routine clinical practice. BMC Med Inform Decis Mak.

[39] Rogal SS, Yakovchenko V, Waltz TJ, Powell BJ, Gonzalez R, Park A (2019). Longitudinal assessment of the association between implementation strategy use and the uptake of hepatitis C treatment: year 2. Implement Sci.

[40] Hartley JF, et al. Employment relations - the psychology of influence and control at work. Br J Ind Relat. 1993;31(4):637.

