# Supplementary material for: Association between the number of adopted implementation strategies and contextual determinants: a mixed-methods study
Source: BMC Health Serv Res. 2022 Dec 13;22:1518. doi: 10.1186/s12913-022-08736-2 (PMC9746001; doi:10.1186/s12913-022-08736-2)
Supplement: Supplementary file 1 — Additional file 1. [file 12913_2022_8736_MOESM1_ESM.pdf]

Aim 1 Interview Protocol (Based on CFIR Guide)

INTRODUCTION

Hi_______________. This is Larry Hearld from the University of Alabama at Birmingham. I'm calling regarding the PCORI IDEAL study. Is this still a good time to talk?

Let me begin by thanking you for agreeing to talk with us today. I know your time is valuable and we appreciate you sharing some of it with us.

Before getting into specific questions, I'd like to start by giving you a brief overview of the project. The purpose of this research project is to evaluate different strategies for implementing an evidence-based shared decision-making aid for patients with lupus, which I will be referring to as the decision-aid throughout the interview. The decision aid is designed to be used by patients to help them make more informed decisions about their lupus treatment options. By our estimates, the decision aid will require about 20 minutes to view by patients, typically on an iPad or tablet or possibly a computer.

Today's portion of the evaluation is a 30 to 45 minute interview regarding your knowledge of the decision-aid and the clinic's current conditions that may support or hinder the implementation of the decision aid. With your permission, today's interview will be recorded, primarily so we can capture your insights accurately. The information that we gather is confidential and no one else outside of the evaluation team can access the specific information you provide. It is our intention, however, to summarize the information provided by you and other people in a report back to clinic leadership for the purposes of identifying the most appropriate implementation strategies for your clinic.

Your participation in today's interview is entirely voluntary. Whether you choose to participate or not will have no bearing on your job or any other work-related matters. You may also change your mind later and stop participating at any time, even if you agreed to participate earlier. Likewise, if you do not want to answer any of the questions during the interview, you can say so and I will move on to the next question. Do you have any questions before we begin?

So before we get into specific questions:

1. Do I have your consent to participate in today's interview?
    a. If no, "thank you for your time".
    b. If yes, do I have your permission to record the interview?
        i. If yes, move on to Background questions.
        ii. If no, turn off recorder.

BACKGROUND

1. **Tell me about your role in the clinic.**
    a. **Probes: What is your title and what are your primary responsibilities in the clinic?**
    b. **How long have you worked in this role? And how long have you worked at the clinic?**

CHARACTERISTICS OF INDIVIDUALS

Next, I want to talk specifically about the decision aid.

1. Based on what you know about the decision aid, what are your initial thoughts about the decision aid? *(Knowledge and beliefs)*

<u>CHARACTERISTICS OF LUPUS DECISION AID</u>

1. As I mentioned in my overview, the decision aid may take up to 20 minutes to be fully viewed by patients, typically on an iPad or potentially a personal computer, during their office visit. What do you think about the length of time for viewing the decision aid? What about showing the decision aid on a tablet in the waiting room? Is that the best approach? What other approach do you think might work in your clinic? *(Complexity)*

2. Does your clinic currently have any shared decision-making programs in place for lupus patients, or has your clinic considered similar types of programs? *(Relative advantage)*
   - If yes:
     a) Can you tell me about this program.
     b) What advantages do you think the decision aid provides compared to the existing program(s), if any? What disadvantages does the lupus shared decision-making aid have compared to existing programs?
   - If no:
     a) Is there any particular reason why your clinic has not pursued these types of programs?
     b) What advantages do you think the decision aid may provide over your existing approach to engaging in decision-making?
     c) What disadvantages does the lupus shared decision-making aid provide over your existing approach to engaging in decision-making?

3. Based on your knowledge of the decision aid, what kinds of changes, if any, do you think you may need to make to the decision aid to make it work effectively in your clinic? *(Adaptability)*
   - Do you think you will be able to make these changes? Why or why not?

<u>OUTER SETTING</u>

1. How do you think the lupus patients served by your clinic will respond to the decision-making aid? *(Patient Needs & Resources)*
   - Probe: Any particular barriers to using the decision aid? *(Patient Needs & Resources)*

<u>INNER SETTING</u>

Now I'd like to shift a little and talk about the clinic more generally.

1. How would you describe the working environment of your clinic? *(Culture)*
   - Probe: If you could describe the working environment in three words, what would those words be? What makes the clinic feel different than other places you may have worked?
   - Probe: Is it formal, with clear cut rules and procedures that people are expected to follow. Is it more informal, like a family, where people are free to be themselves and "just figure things out together"?

2. In general, to what extent do you feel like you and other clinic members can try new things to improve your work processes? *(Learning climate)*
    - Can you tell me about a time when you thought that the usual way of doing things in the clinic might not be the best way. *(Learning Climate)*
        - What lead you to believe the usual way might not be the best way?
        - How did you communicate this concern?
        - How did you create and communicate a new way of doing things?
        - What issues did you run into with other people when trying to create and communicate this new way?
    - Let's flip that question around. Could you tell me about a time when you were asked by someone else to change the way you do something? *(Change readiness)* How did you react?

3. Next I'd like to talk a little about how your clinic generally approaches implementation efforts. *(Learning climate)*
    - Has your clinic engaged in any quality improvement projects to improve patient care in the past two years? How about research projects?
        - If yes:
            1. Can you describe the project. What was the purpose?
            2. Who were the key "players" involved in its implementation?
            3. What was your involvement?
            4. Were people happy with the outcome? What factors helped make it succeed or fail?
            5. In what ways were the clinic leadership involved in the project, if at all? Who?
            6. How well do you think the lessons learned from this project may apply to using the decision aid in your clinic?
        - If no:
            1. Any particular reason why your clinic has not engaged in any of these projects recently?
                1. Probe: lack of resources; no perceived need

    - Probe about implementation in routine care, as opposed to a research study

4. What kind of support would you like to see from leaders in your clinic when implementing things such as the decision aid *(Leadership engagement)*

5. How do you typically find out about new information, such as new initiatives or changes to policies and procedures? *(Networks and Communication)*

6. When you need to get something done or need to solve a problem, who are your "go-to" people? *(Networks and Communication)*

Thinking more specifically again about the decision-aid,

7. From the perspective of clinic personnel, what do you think will be the primary barriers to using the decision aid?
    - Probe: Do you feel like you have sufficient resources to implement and administer the decision-making aid? *(Available Resources)*
        - [If Yes] What resources are you counting on?
        - [If no] What resources will not be available?

8. In your opinion, what kinds of changes to the infrastructure, if any, might be needed to accommodate the decision aid? *(Structural characteristics)*
    - Changes in staffing? Physical layout? Formal policies? Information systems or electronic records systems? Other?
    - What would be needed to make these changes? What would the process look like?

PROCESS

The final set of questions pertain to the implementation process, or how your clinic will go about using the decision aid.

1. In your opinion, who are the key people to get on board with this implementation? *(Opinion leaders)*
    - How do you think they influence others' use of the decision aid?

2. How do you currently communicate with patients?
    - Probe: Direct mailings? Email? Patient portal? Telephone?

3. How do you think your clinic should communicate with lupus patients about the availability of the decision aid? *(Participant/Patient Communication)*

4. How do you think patients should access the decision aid? *(Participant/Patient Communication)*

5. How about clinic personnel. How do you think your clinic should inform clinic staff about the availability of the decision aid and how to use it?

CONCLUSION

1. Is there anything that we haven't discussed today that you think would be helpful for us to know when thinking about implementing the decision aid in your clinic?

Thank you again for taking time out of your busy schedule to talk with us today.
